# Electrically Tunable Optofluidic Metasurface

**DOI:** 10.1021/acsnano.5c18915

**Published:** 2026-03-06

**Authors:** Samuel F. J. Blair, Minahil Khan, Christopher P. Reardon, Steven Johnson, Thomas F. Krauss

**Affiliations:** School of Physics, Engineering & Technology, 8748 University of York, Heslington, York YO12 5DD, U.K.

**Keywords:** tunable metasurface, optofluidics, indium tin oxide, photonics, biosensing, wavefront engineering

## Abstract

The dynamic control of light at the nanoscale has been a longstanding challenge in photonics. More recently, fluidics has been added to the toolkit, leveraging liquid properties as an additional degree of freedom for tunability. Here, we present a tunable metasurface architecture that integrates an optofluidic layer. We use a guided mode resonance platform, whereby a silicon nitride grating is coated with indium tin oxide (ITO) as the active material, operating in the visible-NIR wavelength range (∼800 nm). Our design employs an all-dielectric structure with a fluidic gate to overcome the efficiency-loss trade-off typical of other dynamic metasurfaces. We demonstrate spectral and phase tuning, achieving a near-2π phase shift with a low voltage swing (±3 V), as well as maintaining a strong resonance amplitude (*R* > 80%) with an all-pass filter configuration. These results establish a foundation for high-performance tunable metasurfaces with broad cross-disciplinary applications.

## Graphical abstract

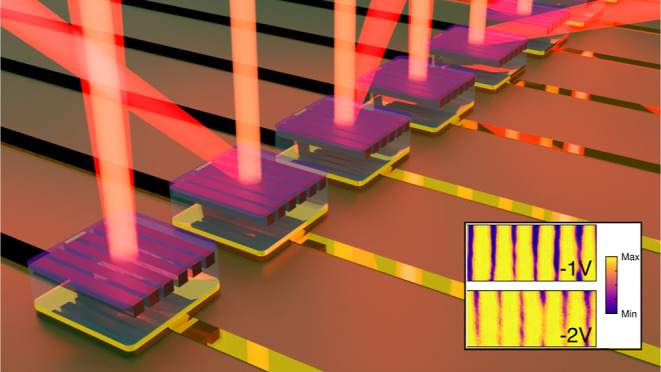

## 1 Introduction

Dynamic control over the properties of light using tunable nanoscale elements has been a long-standing goal in nanophotonics. Metasurfaces, composed of subwavelength resonator arrays, enable precise control of wavefront phase, amplitude, polarization, and spectral characteristics, but typically only in a static fashion; these have been used to demonstrate a wide range of functions, including anomalous reflection, lensing, polarization manipulation, and holography,^1–4^ but the functionality remains fixed postfabrication.

To address this limitation, active metasurfaces are being developed to enable a reconfigurable optical response via external stimuli. Dynamic control can be achieved through spatially multiplexed devices or individually addressable resonators. Early approaches exploited thermo-optic effects^5,6^ or free-carrier modulation in semiconductors such as GaAs and InSb,^7,8^ but these suffered from thermal crosstalk, high power requirements or insufficient phase shift. Other materials such as graphene^9,10^ and lithium niobate^11^ offer particularly high-speed modulation but require high driving voltages and suffer from weak light–matter interaction and correspondingly modest index contrast.

Transparent conducting oxides (TCOs), particularly indium tin oxide (ITO), offer an interesting alternative due to their strong electro-optic response, especially in the epsilon-near-zero (ENZ) regime.^12,13^ Metal–insulator–metal (MIM) structures produce a significant boost in field enhancement, enabling substantial phase modulation.^14–16^ On the other hand, the ENZ nature of the mechanism exposes the signal to significant optical losses. To mitigate optical losses, increasing attention has focused on operating ITO-based devices outside the ENZ regime, particularly in dielectric structures at near-visible wavelengths, where reduced absorption is accompanied by retained tunability. Unlike earlier dielectric platforms that suffered from limited index modulation, the use of ITO now provides a route to overcome this limitation while maintaining high efficiency.

A complementary solution to the tuning problem is offered by optofluidics, which integrates liquids into photonic systems to enable both direct and indirect abstract optical functionalities. For example, optofluidic and soft-matter metasurfaces^17^ have shown promise in applications ranging from low power displays,^18^ optical encryption,^19^ beam steering,^20^ and polarization conversion,^21^ to biosensing^22,23^ and nanoparticle manipulation.^24^ The introduction of fluidics enables the modulation of the refractive index by injecting various liquids or by tuning the electrochemical interaction,^25^ which not only enables dynamic tuning of the metasurface, but also facilitates the light–fluid interaction necessary for biosensing. Optofluidics allows both active modulation and monitoring of chemical or biological processes, combining tunability with unique fluidic functionalities. One particularly promising approach involves electrolyte-gated capacitors (EGCs), where the application of an external bias induces the migration of ions in the fluid to form electrical double layers (EDLs) at the interface with a semiconductor. These EDLs act as ultrathin, high-capacitance layers, significantly amplifying the space-charge region and enabling strong modulation of the carrier concentration and, consequently, the refractive index of the active material.^26^

Here, we combine ITO’s tunability with a fluidic gating mechanism in an all-dielectric metasurface architecture. By coupling an ITO-coated resonant structure with an ionic solution as the gate dielectric, we demonstrate electrically reconfigurable control over both spectral and phase response through the formation of EDLs. This hybrid approach unites the efficiency of solid-state tuning with the versatility of optofluidics, enabling scalable, low-loss, and broadband dynamic metasurfaces.

We exploit the fluidic gating mechanism on a Si_3_N_4_ guided-mode resonance (GMR) grating platform that is coated with a 60 nm layer of ITO (Figure 1), the ITO providing conductivity and electronic control. The ITO surface is then exposed to an ionic liquid, and a voltage is applied between the ITO and a counter electrode immersed in the liquid, as illustrated in Figure 1c. The presence of mobile ions in the fluid is essential to enable EDL formation and modulate the ITO’s space-charge region effectively. This modulation is further enhanced by the nanogap capacitor effect intrinsic to the EDL, enabling dynamic tuning of the optical properties even in the visible–NIR regime, where conventional ITO-based devices typically underperform. Accordingly, the high capacitance of the nanogap EDL at the ITO interface amplifies the charge modulation in the ∼1 nm charge layer, either by increasing the carrier density or by effectively expanding the accumulation thickness. In practice, this produces a unity-order effective refractive index shift in the active later, even at shorter wavelengths.

**Figure 1:**
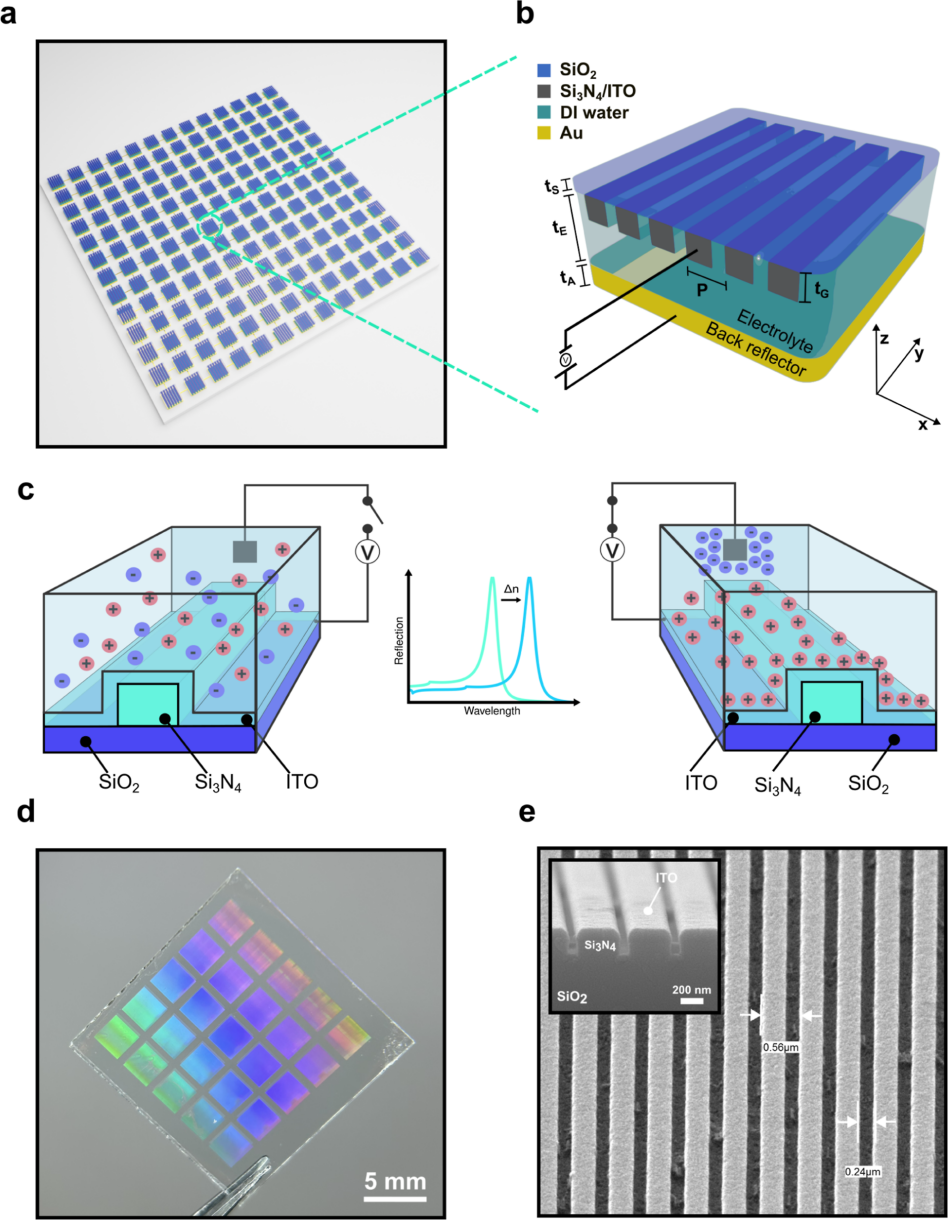
Design, mechanism and fabrication of the optofluidic metasurface. (**a**), Overview, showing an array of metasurfaces that can be individually accessed. (**b**), A single metasurface, displaying the resonance grating, liquid phase and a back reflector to enhance the returned signal. (**c**), Schematic of the tuning mechanism, illustrating how an applied voltage induces a change in the refractive index of the ITO space charge layer through electric double layer formation, shifting the resonant mode of the grating. (**d**), Photograph of a fabricated metasurface. (**e**), SEM micrographs of a grating, showing a top and facet view, displaying the relevant feature sizes.

The electric double layer forms along the ITO surface, while the optical field of the resonant mode is similarly confined near this interface, ensuring a strong interaction between the two. This geometry results in efficient, low-loss refractive index tuning. Together, the optofluidic-tunable metasurface design combines resonant field confinement with electrolyte-enhanced carrier modulation, enabling low-power spectral and phase tunability in a compact and scalable platform.

The devices were fabricated by first etching the nanostructures into a Si_3_N_4_ thin film, before sputter deposition and a high temperature anneal of the ITO layer using the methods detailed in.^27^ Figure 1d shows a fabricated device, together with two scanning electron microscope (SEM) micrographs in Figure 1e of the one-dimensional gratings. We then mount a silicone well to the surface above the patterned region to provide a vessel for the liquids (see Methods for further detail on the device fabrication).

## 2 Results and Discussion

### 2.1 Active Tuning of Spectral Amplitude and Reflection Phase

The operating wavelength range of 780–820 nm was chosen to balance modulation efficiency and optical loss, which scale with wavelength; in the ENZ regime around 1.5 μm, modulation is strong but losses become prohibitive, while the modulation efficiency is too low at shorter wavelength to be practically useful. The grating resonance was designed accordingly, see Figure 2b.

**Figure 2:**
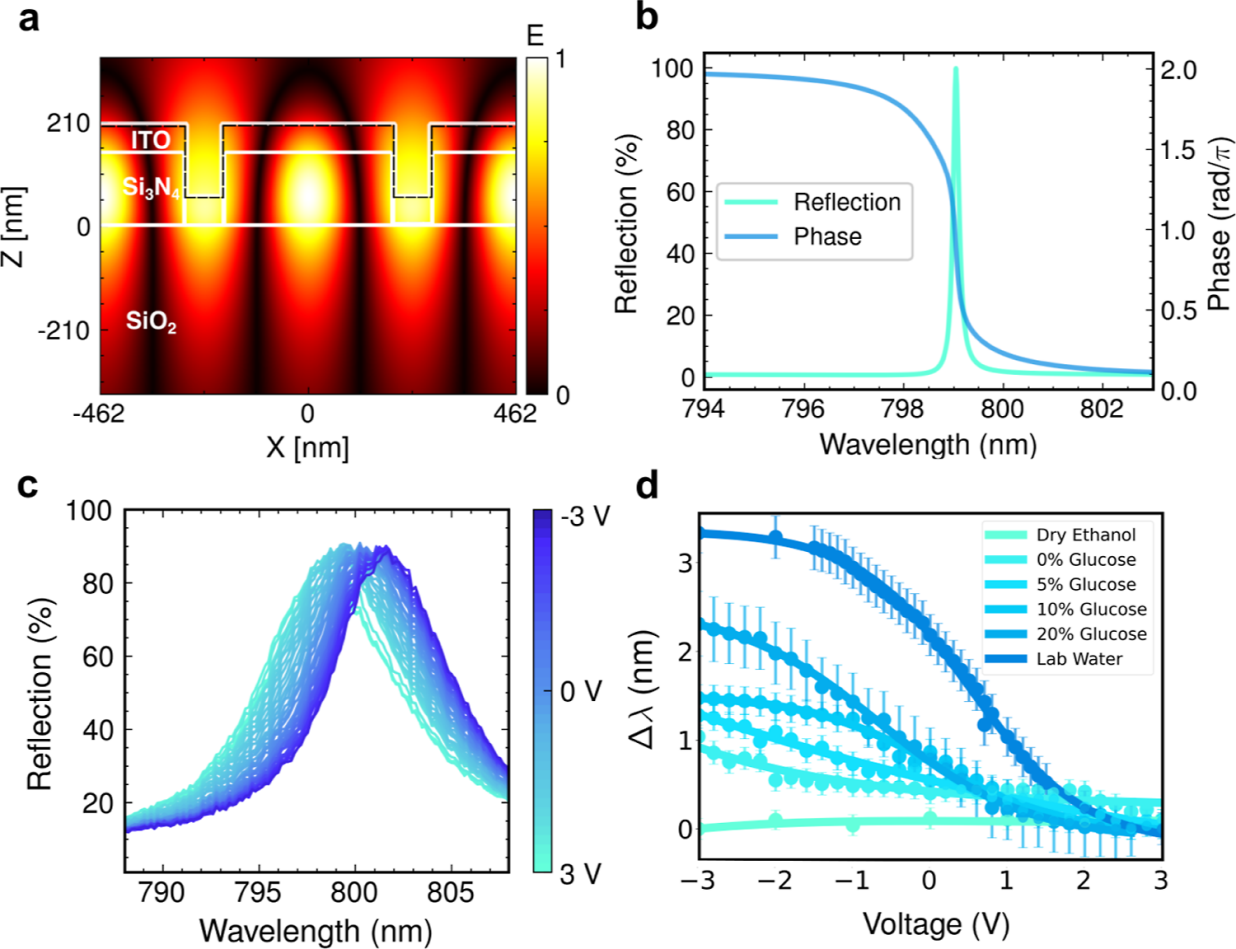
Expected performance of the dynamic spectral modulation. (a), Simulated TE polarized field profile of the grating. The dashed line represents the space charge layer across the top profile. (**b**), Simulated device resonance and phase profile. The parameters are as follows: period = 462 nm, filling factor = 81%, grating thickness = 150 nm, ITO thickness = 60 nm, ITO index = 2.02. (**c**), Experimental spectral modulation data across a ±3 V range. The optical resonance is clearly seen to shift as a function of voltage. (**d**), Glucose dilution series in an ethanol base, including dried ethanol, 0%, 5%, 10%, and 20%, glucose concentrations, and laboratory water as a reference.

The phase response of a GMR arises from two mechanisms-the Fabry-Pérot thin-film and the Bragg resonance-each contributing a potential π phase shift. Hence, tuning of the grating and ITO thicknesses allows control over the total phase shift, where a steep phase response is desirable for low-voltage operation. Through a parameter optimization sweep, we achieve a phase change of approximately 2π over a wavelength range of only 2 nm. Moreover, we note that this design also achieves a strong overlap between the space-charge region and the optical mode (Figure 2a), which is advantageous for efficient modulation.

Next, to better understand the underlying mechanism, we modeled the effective index modulation induced by the space charge region, using FDTD methods.^28^ The electrolyte enhances modulation through the formation of the electric double layer (EDL), which increases the interfacial charge carrier density that amplifies the effect of the space charge layer on the optical mode. An analytical electrostatic model, combined with a Drude dispersion framework $\left(\epsilon \left(\omega \right)=\epsilon_{\infty }-\frac{\omega_{p}^{2}}{\omega^{2}+i\omega \Gamma }\right)$ , was used to relate the applied voltage to the index modulation. The following parameters were used:^27^ ϵ_∞_ = 3.9, *m*_e_^*^ = 0.5m_e_, Γ = 3.9 THz, *N* = 1.25 × 10^25^ m^–3^, and λ = 800 nm. The EDL enhancement effectively generates a new, amplified dispersion curve for the ITO across the narrow spectral region the device operates at, with a significantly steeper gradient $\left(\frac{\Delta \epsilon_{r}}{\Delta \lambda }\right)$ . The resulting index change was incorporated into optical simulations to quantify spectral and phase shifts. The model predicts a unity-order index modulation in the ITO (accumulation/depletion) layer with a ±1 V bias, supported by the EDL-induced field enhancement (Supporting Information, Figure S3a). This results in a resonance shift of ∼4.5 nm and a full 2π phase shift across the spectral window. Further details on the simulations can be found in the Supporting Information (Section 3).

Next, we explore the active properties of the device experimentally. We achieve active modulation by applying a voltage between the ITO layer and the reference electrode immersed in the electrolyte. This voltage forms a space charge layer at the ITO-electrolyte interface layer, thereby electrically tuning the refractive index of the ITO and inducing a shift in the device’s resonant wavelength. The devices were measured in an electrolyte-filled culture well, with a platinum wire pseudoreference electrode and the ITO contact as ground. A positive bias led to electron drift toward the ITO interface, decreasing the refractive index and blue-shifting the resonance; negative bias produced the opposite effect. Using ultrapure water as the electrolyte, a total resonance shift of Δ3.5 nm was observed with a voltage swing of ±3 V, with peak positions extracted using Fano fitting. The response followed a sigmoidal voltage dependence, saturating beyond ±3 V. The tuning was asymmetric, with greater blue-shifts under positive bias, consistent with the known disparity between accumulation and depletion in ITO. Looking deeper into the mechanism, it is useful to consider the possible contributions from any electrochemical effects of the fluid. We note that the device exhibited a change in capacitance with voltage, which we associate with the double layer formation. We also saw no hysteresis in the photonic response to voltage, which suggests that the effect is purely capacitive rather than related to a redox reaction.

We note that the effect we describe is equivalent in magnitude and operates at a lower voltage than ITO ENZ devices previously described in the literature.^13,16,29^ We hypothesize that two possible mechanisms might be responsible for this remarkable refractive index shift, (a) ion diffusion or (b) alignment of polar molecules. To pinpoint the mechanism, we investigated a range of electrolytes, summarized in Figure 2d. All water-based liquids exhibited a strong response, but water is both polar and an ionic conductor; even ultrapure deionized (DI) water tends to have a residual ion content. We therefore tried ethanol, which is a nonionic (but polar) fluid. Interestingly, ethanol exhibited no modulation effect. When we then added increasing concentrations of a glucose-water stock solution to the ethanol, the modulation effect recovered. This observation suggests that polarity (mechanism b) is not behind the large index shift, whereas ion content (mechanism a) is. Subsequent doping of ethanol with a glucose-water stock solution allowed precise control of the ion concentration and thus a tuning of the device. Notably, the laboratory water exhibited the strongest modulation, attributed to its contaminated nature and residual ion content. The glucose dilution series was conducted using a systematic protocol to test for hysteresis. Measurements were taken at each voltage (±2 V, ±3 V, etc.) and returned to 0 V between each step. The consistency of the response upon returning to 0 V after each voltage excursion, combined with the tight error bars obtained from repeated measurements at each condition, demonstrates negligible hysteresis. Had hysteresis been present, a systematic drift in the baseline response or deviations between forward and reverse voltage sweeps would have been observed. This behavior aligns with related systems.^30^

These results indicate that the observed modulation is ion-driven. This also explains the saturation of the resonance shift at approximately ±3 nm, which arises from Debye screening-the electrostatic shielding that limits electric field penetration and suppresses further carrier accumulation at the double layer.^31,32^ The dominant modulation mechanism is therefore the formation of an ionic double layer at the ITO interface. Upon application of a voltage, ions in the liquid accumulate at the electrochemical double layer, while charge carriers accumulate in the ITO. In a symmetric system, the accumulation of positive charge on one side of this capacitor would produce an optical response that is similar in magnitude, but opposite in sign, to that produced by negative charge accumulation on the other side, resulting in minimal net modulation. In the optofluidic system, however, the charge carriers are fundamentally different: the ions in the liquid possess a much larger effective mass than the electrons in the ITO.

Within the Drude model, the contribution of a charge carrier to the permittivity scales with the plasma frequency, ω_*p*_^2^∝e^2^/m. Consequently, electron accumulation or depletion in the ITO dominates the optical response. The small electron mass leads to a large change in plasma frequency and thus a strong modification of the permittivity, whereas the high-mass ions contribute negligibly. Despite overall charge neutrality at the interface, electrons therefore exert a disproportionately large influence on the effective index of the resonant mode compared to the ions in solution. As a result, the modulation induced on the ITO side is not counterbalanced by an equivalent opposing effect in the liquid, which explains the strong optical modulation observed.

Having understood the fundamental mechanism, we proceeded to measuring the voltage-dependent phase response, using interferometry. Figure 3a shows a representative interferogram and the selected regions of interest (ROIs) used for analysis. Interference fringes were extracted as row-wise intensity profiles and fitted with Gabor functions, which provided a better fit than a simple sinusoidal fit by accounting for both the oscillatory behavior and the spatial localization in the fringe pattern.

**Figure 3:**
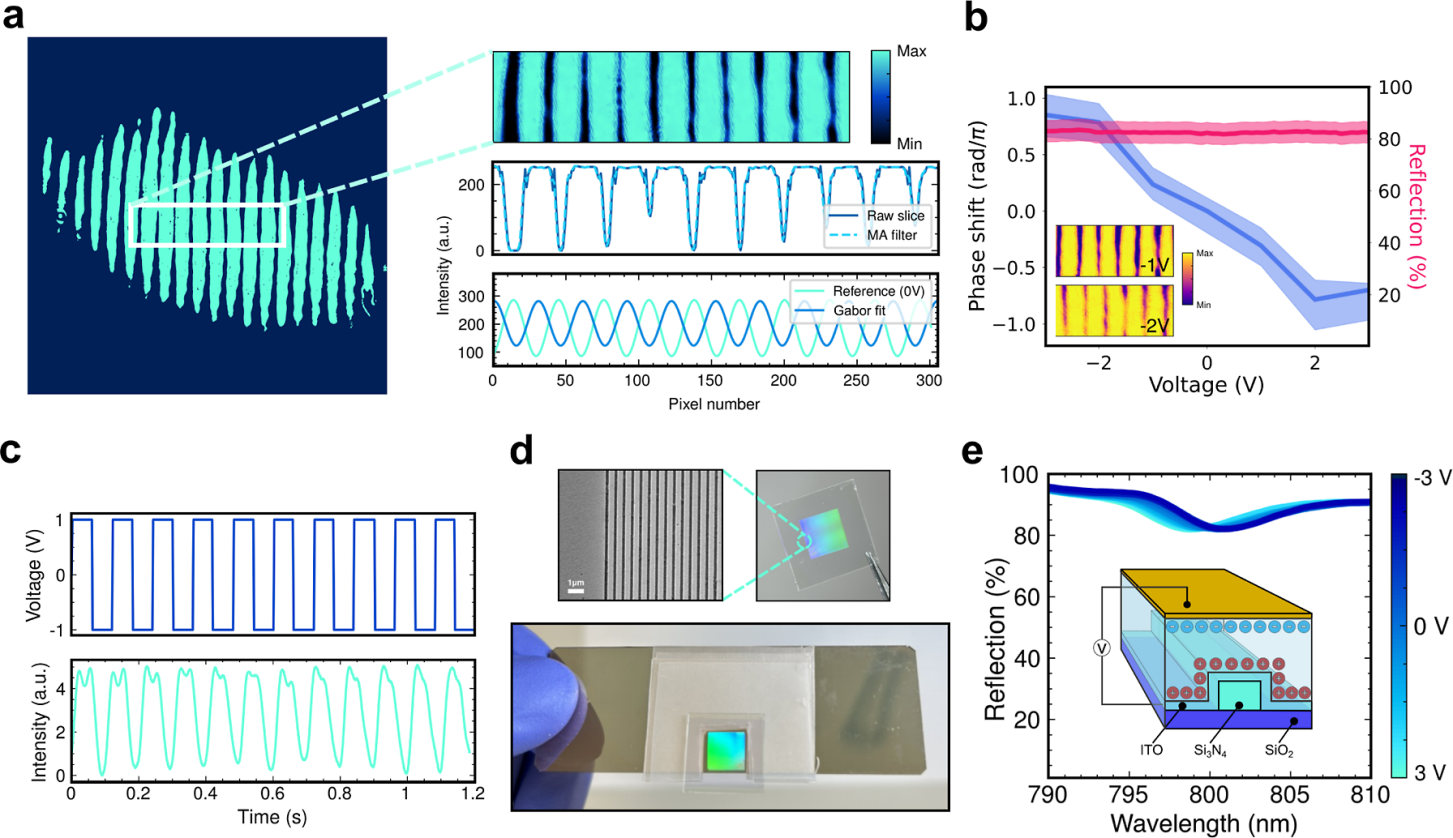
Dynamic phase tuning and all-pass filter capability. (**a**), Example interferogram data with extracted phase information. An ROI and corresponding slice from the interferogram are shown. (**b**), All-pass reflection and phase as a function of voltage. (**c**), Reflected intensity variation with cyclic voltage. Switching speeds of 30 Hz are achievable. (**d**), SEM micrograph and two photographs of the fabricated device, with the bottom photograph showing the bonded device. (**e**), Spectral modulation, achieving an approximately 4 nm resonance shift across a ±3 V range. A schematic of the all-pass device is shown in the inset.

To improve statistical accuracy, four slices per ROI were analyzed and fit results aggregated using a cumulative distribution function (CDF), allowing extraction of the median phase and interquartile range. This process was repeated across four ROIs per voltage setting. All voltage steps were applied in a randomized order to mitigate the influence of any mechanical drift (see Supporting Information for further details). Figure 3b shows the complete phase data set, where the extracted phase shift is relative to 0 V. Across a ±3 V range, we observe a voltage-tunable phase shift of 1.75π ± 0.1π. The error bars associated with the phase data stem mainly from the mechanical drift and vibrational noise of the interferometer across multiple repeat measurements. The uncertainties could be reduced through noise filtering the data or by implementing a full mechanical isolation stage. We note a mismatch between our simulated and experimental results in terms of achieved phase shift and attribute this disparity to the lower experimental quality-factor (*Q*-factor) of the optical mode. Here, the reduced quality increased the total losses in the system, exhibiting itself as a larger voltage requirement per phase increment. Figure 3c shows the switching speed of the device. We used a square-wave voltage and monitored the reflected intensity at a fixed wavelength of 780 nm. We observed a modulation bandwidth of ∼30 Hz, which was independent of ion concentration.

### 2.2 Tunable All-Pass Filter

A key challenge in tunable metasurfaces is the unwanted amplitude modulation that usually accompanies phase tuning across a resonance, resonances being often used to enhance the low refractive index modulation by steepening the phase response. Unavoidably, the same tuning mechanism also modulates the reflectance amplitude, which is evident from Figure 2. To address this limitation, we implemented an all-pass architecture by bonding the device to a gold back reflector. The idea is simple: on resonance, the light is reflected by the GMR, while off-resonance, the light is reflected by the gold mirror using a ∼400 μm spacer.

We built a suitable holder to accommodate the sample, fluidic channel and top mirror. The fluid electrolyte was injected and then encapsulated in this structure (Figure 3d). Optical measurements showed a significantly improved reflectance band across the ±3 V voltage range (Figure 3e). The results have been overlaid with the phase shift data in Figure 3b to highlight the device’s ability to achieve a modulated phase at constant amplitude. Here, the reflection values have been extracted at a single wavelength, allowing for a near flat reflection band across a few volts, or spectrally a few nanometres. The spectral dip in the reflectance spectrum is a result of an imperfect grating fabrication, leading to a less than 100% reflection at resonance, and some absorption and scattering loss from of the ITO. Fabrication protocol improvements would likely reduce or remove the dip. The baseline reflection of approximately 90% is due to carbon and other impurities in the thin Au film incorporated during evaporation, increasing the insertion loss of the device. Moreover, the loss is readily improved by increasing the Au film purity. As the wavelength shifts of the resonance in either direction are small, the reflectance value remains around the flat band at the resonance wavelength (800 nm). The device was designed to operate at the special instance where all of the spectra at different input voltages overlap, and this effect can be seen at 800 nm in Figure 3e. This effect results in a < 2% change in the reflectance across this tuning range. Hence, combining this all-pass approach with the phase tuning results yields a 1.75π phase shift with a small amplitude variation over the ±3 V operating range. Overall, the experiment highlights that resonant enhancement can provide efficient phase tuning without paying the penalty of amplitude variation.

The operational speed of the device naturally emerges as a key point of discussion. The optofluidic component reduces the modulation speed to levels lower than GHz-speed traditional ITO modulators. However, many applications, such as tunable lenses, displays, adaptive optics in astronomy, or slow time scale biosensing, do not require high-speed modulation and benefit instead from the device’s low complexity, power consumption, and noise. To improve speed where needed, a high conductivity electrolyte solution would produce a faster switching speed as $\tau ∼\frac{1}{\sigma }$ where τ is the polarization time and σ the conductivity. Hence, ion gels offer a promising solution due to their high conductivity (>1 mC/cm), enabling modulation in the >100 kHz range^33–35^ and compatibility with solid-state systems. With this improvement, the device could then target high-speed applications, such as LiDAR or static-weight neural-networks, which would benefit from the modulation depth the device achieves at 800 nm. We also note that the volatile nature of the EDL-based modulation generates fouling of the device surface at high voltage biases. Nonetheless, this effect is an acceptable trade-off given the enhanced modulation depth we observe for the device. Since the device readily achieves a near 2π phase shift across a low magnitude voltage window, degradation remains minimal as high voltages are not required to achieve substantial optical modulation.

### 2.3 Experimental Demonstration of Biosensing

To illustrate a second application of the device that exploits its unique optofluidic capability, we configured it as a simple biofilm detection sensor. For the avoidance of doubt, this configuration uses the amplitude response of the resonance, without the all-pass gold mirror.

We first calibrated the voltage vs refractive index response with a glucose dilution series before sensing*Escherichia coli* bacteria colony formation on the sensor surface. The voltage required to tune the resonance back to its peak was extracted from brightness–voltage curves as a function of refractive index, enabling quantitative refractometric sensing. A shift in the medium’s refractive index modifies the effective index of the guided mode, thereby changing the voltage required to return the resonance to its original resonant wavelength.

We first tested glucose solutions of 0 to 15% in 5% increments, diluted 3:1 in deionized water, yielding a refractive index range of 1.333–1.334 RIU (measured with a commercial refractometer). For each concentration, the grating was illuminated with a monochromatic source and the voltage was swept from −3 V to +3 V to find the maximum reflectance. This method produced a linear voltage–index calibration curve with a sensitivity of 630 ± 56 V/RIU (Figure 4a).

**Figure 4:**
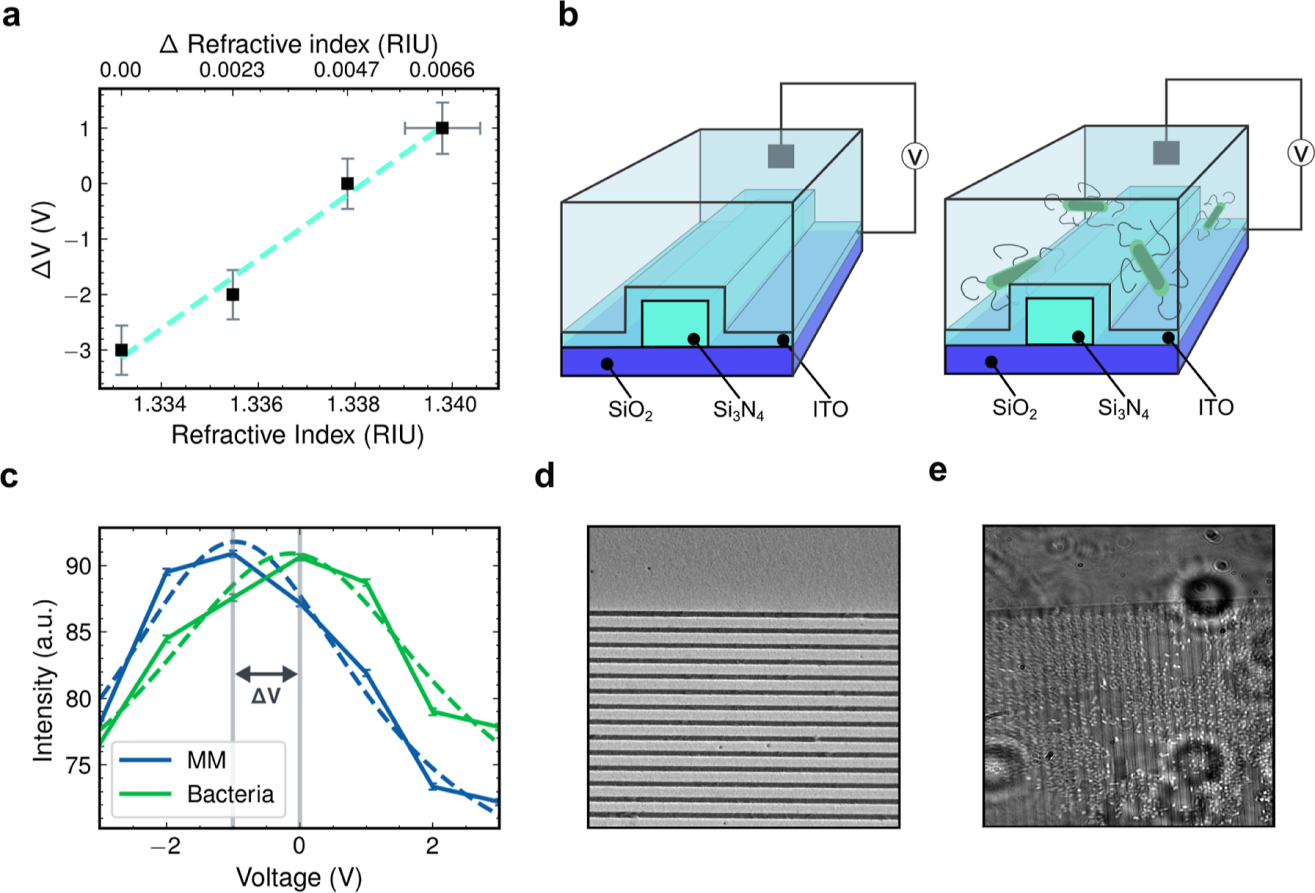
Biosensing demonstration. (**a**), Dilution series calibration, showing the voltage required to compensate a given refractive index shift. (**b**), Schematics of the device with and without bacteria in solution. (**c**), Traces of the resonant wavelength of the device with media and a strain of *E. coli*. (**d,e**), SEM micrographs and photographs with and without bacteria. The periodic structure of the grating can be identified, with the small white spots indicating the bacteria.

To contextualise this result, we compare it with an analogous chirped photonic crystal sensor based on the device presented by Triggs et al.,^36^ which exhibited a spectral sensitivity of approximately 137 nm/RIU. We note that other geometries can achieve significantly greater sensitivities, for example upward of 500 nm/RIU.^37^ However, we are able to make a direct comparison to our Si_3_N_4_ GMR based device without the ITO layer that facilitates the electrochemical effects. Hence, the 630 ± 56 V/RIU of our optofluidic sensor converts to a sensitivity of approximately 368 nm/RIU, providing a marginally enhanced sensitivity in comparison to an ordinary GMR based sensor. This conversion was calculated by multiplying the sensitivity from Figure 4a (V/RIU) by the spectral shift per volt (nm/V), extracted from Figure 2d (3.5 nm/6 V). We suggest that this small enhancement may arise from the dynamic electrochemical modulation at the ITO surface, whereby the electrolyte ions modify the surface charge distribution and thereby the electrostatic properties of the EDL. This effect could result in an amplification of the device sensitivity beyond conventional evanescent-field effects. Having measured the sensitivity, we can next determine the limit of detection (LOD), which is typically defined as three times the standard deviation (σ) of the noise. To evaluate the LOD, we toggled the voltage between +1 V and +2 V over 20 cycles and measured the statistical fluctuations in the extracted resonance voltage (see Supporting Information). The measurements yielded a 3σ of 0.0696 nm. Based on the refractive index sensitivity (368 nm/RIU), we calculate an LOD of 2.0 × 10^–4^ RIU, which is comparable to passive GMR sensors.^38^

Finally, we turn to a biosensing demonstration via the detection of*E. coli (E. coli)* (strain MG1655) suspended in a M9 minimal media (MM) (Sigma-Aldrich) growth environment on the sensor surface. After allowing a time period for the cells to settle on the device (∼15 min) the resonance voltage was measured and compared to the signal from the media alone. The experiment with only MM provides the “nominal” condition, allowing us to draw conclusions from the measurement with*E. coli* to remove any drift or ion adsorption artifacts that may occur during the settlement window. Here, the difference (in voltage) between the two peaks is equivalent to the partial coverage of the bacteria. Figure 4c depicts the brightness traces as a function of voltage of the two conditions. A clear voltage shift of 1.02 ± 0.03 V was observed, corresponding to a refractive index increase of 0.0016 ± 0.0008 RIU. Given the*E. coli* body size (∼1 μm), only the portions of the cells within the evanescent field (∼200 nm decay length) contribute to sensing. No further index change was observed beyond the initial 15 min incubation, consistent with surface–level interaction only. The resonance shift may also be partly attributed to the change in surface potential or double-layer capacitance as a result of the adsorption of the negatively charged bacteria on the ITO electrode. Here, the bacteria could stimulate a diffusion of free carriers in the ITO charge layer, although this effect would likely be negligible as the Debye screening length in media is small (∼1 nm)^39^ in relation to the bacteria size (∼1–2 μm). Nonetheless, the device is able to detect the bacteria through the small refractive index change, measured in voltage. This result aligns well with other studies that explore biofilm growth as a function of resonance shift,^40,41^ ultimately placing the magnitude of the observed shift into context. These results demonstrate that the optofluidic metasurface achieves sensitivities and LODs comparable to existing state-of-the-art biosensors while exploiting a novel modality. Ultimately, the device functions as a compact, label-free biosensor, capable of detecting refractive index shifts on the order of 10^–4^ RIU using monochromatic illumination and voltage control alone.

The device’s modulation depth arises from the resonant mechanism of the GMR, and is therefore intrinsically wavelength dependent. This resonance, however, enables the large phase excursion observed, owing to the high quality factor of the mode. Moreover, we note that the resonance line width remains broader than that of many high-Q sensing platforms, such as ring resonators, providing a comparatively larger operational bandwidth. In contrast to plasmonic and other resonant tunable metasurfaces, the present device maintains high transmission amplitude while achieving substantial phase modulation, reducing insertion losses and broadening potential application space. Moreover, unlike conventional liquid-crystal-based phase modulators, the optofluidic nature of the platform enables the liquid phase to carry biochemical functionality, introducing an additional degree of freedom that extends applicability toward biosensing and biointegrated photonic systems.

Unlike other tunable optofluidic systems such as liquid crystals, which rely on the liquid for modulation, our device uses the fluid as an extra degree of freedom to expand measurement capabilities. This fluidic metasurface can directly interface with biological environments, enabling applications in implantable bioelectronics or beam steering for minimally invasive imaging.^42^ Current optogenetic fiber probes integrate electrodes for recording and stimulation but lack precise light modulation.^18,43,44^ Our metasurface could be integrated at the fiber tip for dynamic light control in optogenetics and tunable-focus bioimaging.^45,46^ Fiber-integrated variants could also act as pH sensors or refractive index detectors, offering compact, low-power, and reconfigurable photonic interfaces for biological systems.

## 3 Conclusions

This work demonstrates the dynamic control of light through an electrically tunable optofluidic metasurface. We achieve a near-2π shift in the reflection phase across an ultralow voltage swing of ±3 V at constant reflection. While modulation of the real part of the refractive index is generally low in the visible wavelength range across the majority of tunable materials, our results prove that significant index tuning of ITO is possible away from the ENZ regime through an optofluidic enhancement. Tunable devices often face a trade-off between the modulation depth and the applied voltage; our results overcome this barrier through optofluidic amplification. Other solid-state ITO devices rely on ENZ conditions or highly confined plasmonic structures, but these may incur significant losses and reduce resonance amplitude or modulation efficiency, or both. Conversely, our device exhibits low optical losses inherent to its dielectric design. When integrating an all-pass filter with a back-reflecting mirror, the device attains nearly 100% efficiency across a wide spectral range. Through the optofluidic approach, we realize ENZ-like tuning with high efficiency and low voltage, overcoming the limitations of the plasmonic approach.

These metrics position the optofluidic metasurface as a promising platform for emerging applications that require low voltage phase control of visible light, especially in comparison to similar tunable devices across a variety of modulation mechanisms.^47,48^ One potential use case is addressable holography displays,^49^ where precise and tunable wavefront shaping is essential. Similarly, the device could serve as an active color filter for display technologies that must adapt to varying ambient light conditions.^50^ Its compatibility with standard CMOS voltage levels (0–3.3 V) and capacity for high-density patterning make it well-suited for integration into next-generation photonic systems.

## 4 Methods

### 4.1 Sample Fabrication

We start the fabrication with a 15 × 15 mm cleaved chip of 150 nm Si_3_N_4_ on SiO_2_ (Silson). The sample is then cleaned in a Piranha solution (3:1 H_2_SO_4_: H_2_O_2_) for 10 min to remove any organic residue, before a cascade rinse in deionized (DI) water, followed by acetone and IPA rinses in a sonic bath. The AR-P 6100.13 (AllResist) electron-beam resist was then spin coated onto the Si_3_N_4_ surface at a speed of 6500 rpm for 45 s and a bake of 3 min at 90°C and then 180°. An AR-PC layer was next spin coated onto the chip at a speed of 4500 rpm and baked for 2 min at 90°C to act as a charge dissipation layer. The photoresist thickness was approximately 2 μm, as measured by surface profileometry. The resist was next patterned using electro-beam lithography (Voyager system, Raith GmbH) using a beam current of 0.6 nA at an aperture diameter of 40 μm, which produced a base dose of approximately 145 μC/cm^2^. After exposure, the sample was first rinsed in DI water for approximately 20 s to remove the AR-PC layer, before a development in Xylene for 2 min and a rinse in IPA solution. Pattern transfer was achieved by reactive ion etching (RIE) on a bespoke in-house system. We used a flow ratio of 58:2 of fluoroform (CHF_3_) to oxygen (O_2_) at a pressure of 0.18 mbar and DC bias of 370 V, to achieve a 150 nm etch in 7 min. After the dry etch, the remaining resist was stripped in 1165 (MicroResist) for 12 min at 60°C, followed by acetone and IPA for 5 min.

To deposit the ITO layer, we used a DC magnetron sputtering system with an ITO target of a 90/10 weighting of In_2_O_3_/SnO_2_. Samples were rotated during deposition at a pressure of 7.5 × 10 ^–3^ mbar, a power of 100 W and a rate of 0.4 Å/s. An Ar gas flow of 20 SCCM was used with zero oxygen flow to maximize the oxygen vacancy contributions to the carrier density. Samples were also left to cool in the chamber at vacuum overnight to further maximizing the vacancy contribution by avoiding adding impurities to the films while warm. Finally, samples were annealed at 500°C for 1 h, with a ramp of 5°C/min in an Ar environment to relax defects and remove impurities, further increasing the ITO conductivity.

To fabricate the all-pass filter device variant, a glass slide was coated in Cr and Au layers (5 nm/100 nm) to act as the back reflector for the all-pass variant of the tunable metasurface. A slot was next cut out of a parafilm spacer, allowing an aperture for the patterned area of the chip. The chip was then bonded to the parafilm-back reflector stack by heating the device for 2 min at 100°C, forming a capillary. Liquid was then applied in the capillary, before being sealed by the parafilm with a repeat of the above process. Electrodes were then electrically attached to both the backreflector and ITO surface using conductive epoxy paste.

### 4.2 Spectral Modulation Measurements

To analyze the spectral characteristics of the device, we used a reflection set up that operates under Köhler illumination (Supplementary Figure S7). Unpolarized light from a halogen source (Thorlabs SLS201L) passes through a linear polarizer, before a Köhler lens that is focused to the back–focal plane of the objective (Olympus 4x PLN (NA = 0.10)) to ensure adequate collimation of the beam. The sample is then illuminated with the reflected signal from the sample then passing back through the objective before a beam splitter to feed a spectrometer (Thorlabs CCS175 Compact spectrometer) and camera (Thorlabs DCC1545 M CMOS camera). All the reflection spectra are normalized to the reflected signal of a silver mirror.

For electrical measurements, an electrical probe (FormFactor DC Probe Probes DPP105-M-AI-S) was applied to the ITO to pass a voltage across the device using a DC power supply. A grounded counter electrode (platinum wire) was inserted into the fluidic elements for modulation measurements to apply a bias across the device. Culture wells were used as a vessel for the fluid above the sample.

### 4.3 Phase Tuning Setup

We characterized the phase of the device using a bespoke benchtop interferometric setup designed to minimize vibrational noise (Supplementary Figure S8). The system employed a collimated 786 nm laser module (Thorlabs CPS780S), a 50:50 plate beam splitter (Thorlabs CCM1-BS014/M), a silver mirror (Thorlabs PF05-03-P01), and a USB CMOS camera (Thorlabs DCC1545M). The reference mirror was mounted on a kinematic stage for angular adjustment and a rail slider for optical path length tuning. A lens was used to aid in on-chip beam alignment before being removed to take measurements. Phase information was extracted from the interferograms of the active tunable metasurface by analyzing intensity variations across selected image slices as a function of voltage.

### 4.4 Switching Speed Measurements

The modulation speed of the device was characterized using a fixed-wavelength laser illuminated a grating (Thorlabs CPS780S), and the reflection intensity at resonance was monitored. An alternating square wave voltage of ±1 V was applied to the device using a signal generator (GW INSTEK GFG-8219A), inducing cyclic changes in the phase and reflected intensity. Here, the voltage induced a refractive index change in the ITO, which shifted the GMR resonance position away from the laser wavelength. This effect produced a drop in the intensity, that was detected with a photodetector (Thorlabs PDF10A/M). This approach thus allowed a measurement of the temporal response as a function of applied voltage. Modulation speed was ultimately determined by analyzing the amplitude variation of the resonance.

### 4.5 Simulations

Analytical simulations were carried out using rigorous coupled-wave analysis (RCWA) to investigate the influence of grating parameter variations on the optical mode characteristics. Parameter sweeps enabled identification of grating configurations yielding suitable phase profiles. Full-wave numerical modeling of the nanostructures was performed using the finite-difference time-domain (FDTD) method implemented in commercially available software (Ansys Lumerical FDTD Solutions). The 1D periodic grating was modeled as a 150 nm-thick Si_3_N_4_ layer with a uniform refractive index of 1.89 and negligible absorption. The ITO layer was treated as a continuous film, with uniform thickness on the grating tops and troughs, while the sidewall thickness was varied as a percentage of the total ITO thickness. Photonic and electrical parameters of the ITO were taken from previous literature,^27^ with a refractive index of 2.05. A 1 nm charge accumulation layer at the ITO–electrolyte interface was included by locally modifying the refractive index. Simulations assumed plane-wave illumination and periodic boundary conditions on all sides. For charge-layer calculations, the vertical mesh step was set to d_*z*_ = 0.2 nm to accurately resolve the thin layer.

## Supplementary Material



## Notes

Details on metasurface fabrication, structural and dynamic simulations and modeling, ITO dispersive effects on modulation, Full reflection phase experimental data, optical measurement set ups, device switching speed measurements, biosensor limit of detection (LOD), achievable phase modulation (PDF)
