# Supplementary material for: Electrically Tunable Optofluidic Metasurface
Source: ACS Nano. 2026 Mar 6;20(10):8461–70. doi: 10.1021/acsnano.5c18915 (PMC13001100; doi:10.1021/acsnano.5c18915)
Supplement: Supplementary file 1 [file nn5c18915_si_001.pdf]

# Supporting Information

# Electrically Tunable Optofluidic Metasurface

Samuel F. J. Blair, Minahil Khan, Christopher P. Reardon, Steven Johnson, and Thomas F. Krauss*

*School of Physics, Engineering & Technology, University of York, York YO10 5DD, United Kingdom*

E-mail: samblair_@hotmail.com,thomas.krauss@york.ac.uk

# 1 Metasurface fabrication

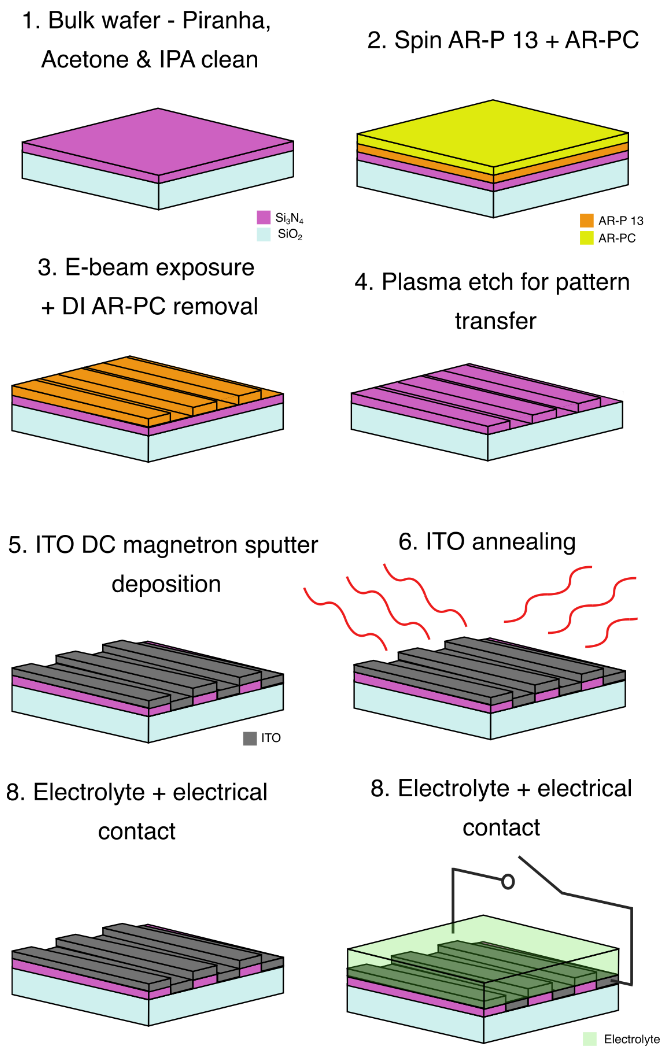


**Figure S1:** Fabrication process of the tunable metasurface, including an electron beam lithography write, reactive ion etching and thin film deposition of ITO.

# 2 ITO geometrical simulations

Fig. S2 shows simulated spectral and phase maps of the optofluidic metasurface for increasing ITO thickness. Here, an 80% filling factor was used to grant a suitable Q-factor and phase response. The sputtered ITO is also a non-conformal coating. To more accurately model the structure, we used a grating sidewall thickness of 50% of the total ITO thickness that was obtained from analysis of SEM micrographs of grating facets. As a result, the mode begins to disappear for thicknesses greater than around 60 nm as the gap in between subsequent ridges begins to fill with ITO. For the mode to be sustained, a region of air is required to meet the index distribution required for resonance. Moreover, thicker layers of the higher index ITO will also provide an increased Q-factor. Hence, for our device, we chose to operate with a 60 nm ITO layer to balance the Q-factor and tolerance trade-off.

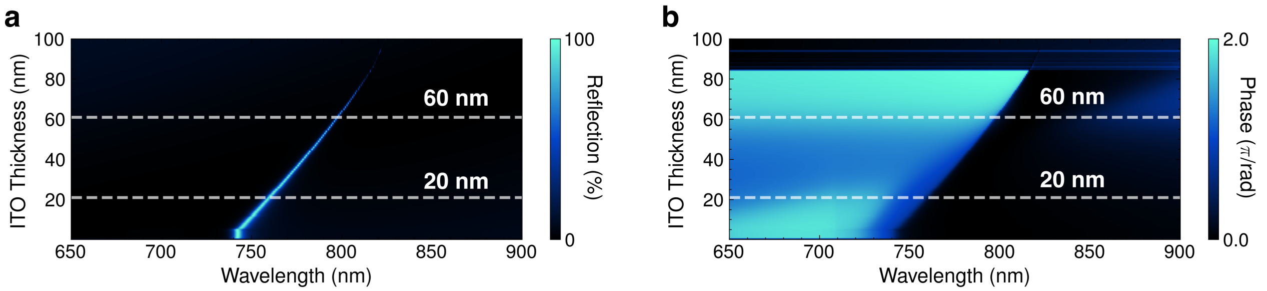


**Figure S2: Metasurface ITO thickness variation.** Spectral (**a**) and phase (**b**) maps for increasing ITO thickness from 0 to 100 nm.

# 3 Dynamic modulation modelling

To model the dynamic behaviour of the active charge region, a numerical FDTD simulation was used to accurately resolve the index change in the small space charge layer. Previous work on ordinary solid-state ITO tunable devices has shown the space charge region to be of the order of 1 nm.[1] However, in the optofluidic device detailed here, the ion migration in

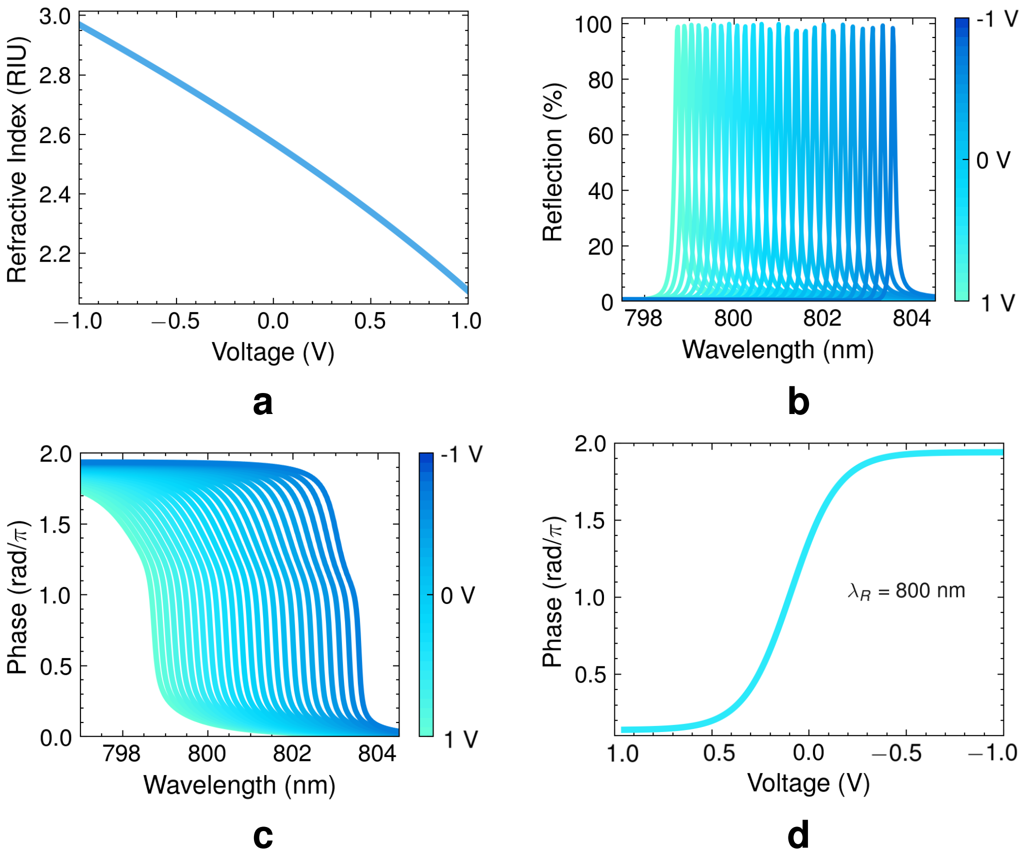


**Figure S3: Modulation performance of the tunable metasurface.** **a**, Refractive index modulation in the 1 nm ITO layer as a function of voltage, determined from first principle parallel plate capacitor equations. Reflection **b** and phase **c** modulation across a ±1 V range. **d,** Relative phase shift as a function of voltage, extracted from **c.**

the fluid and the conductivity enhancement of the space charge layer produce a complex system that is difficult to model to a highly accurate degree. Nonetheless, the structure can still be viewed as a simple effective index change, regardless of the magnitude or effect of the field enhancement. For example, it is irrelevant if the thickness of the space charge region is increased in reality, or if the range over which the index fluctuates varies; both of these effects induce an effective index change to shift the resonance. As a result, we chose to model a 1 nm active region along the top perimeter of the device, with a unity order index variation to provide a sensible effective index change of the unit cell.

We next approximated the index shift per unit voltage by modelling the device as a solid-state capacitor but with a field enhancement factor. The Drude parameters are ex-

tracted from the dispersion curves formulated in ref[2] ($\epsilon_\infty$ = 3.9, $m_e^*$ = 0.5$m_e$, $\Gamma$ = 3.9 THz, N = 1.25 $\times$ $10^{25}$, $\lambda$ = 800 nm), and the field is amplified by increasing the DC permittivity and space charge layer thickness to model an effective increase in the electric field that is induced by the EDL capacitance. Here, the DC permittivity is treated as an effective tuning parameter, used to reproduce the field amplification expected under EDL-dominated conditions. As the enhanced modulation mechanism arises from EDL amplification, a conventional dispersion curve for this “enhanced” ITO is non-trivial, and would only represent an “effective” response over the narrow wavelength band of device operation, as is essentially represented by Fig. S3a. Hence, a unity order index shift within the space charge layer was found to be achievable with a ± 1 V bias (Figure S3a). The voltage induces an order of magnitude increase in the carrier density within the space charge region (1.25 $\times$ $10^{19}$ to approximately 1.4 $\times$ $10^{20}$). This high level of modulation is possible due to the estimated amplification from the electrolyte double layer. The voltage and index results are finally fed into the FDTD model to approximate the reflectance and phase shifts as a function of voltage, displayed in Figures S3b and S3c, respectively. Thus, the device achieves a resonant wavelength shift of approximately 4.5 nm with a ±1 V bias, and a $2\pi$ phase shift across a 2 nm wavelength band.

# 4 ITO dispersion effects

To further test the modulation mechanism of our optofluidic metasurface, we conducted modulation tests are two different wavelengths. According to theory, the modulation efficiency is worse at visible wavelengths as the ITO dispersion curve is less steep. Hence, a greater stimulus is required in order to obtain the same index modulation as at NIR wavelengths. This fact is why the majority of research on ITO operates at the steepest part of the dispersion curve at the telecom range, which also coincides with the ENZ point. An experimentally determined ITO dispersion curve is depicted in Fig. S4a.

This is indeed the trend we observe, with experimental modulation data shown in Fig. S4b for devices optimised to operate at 646 nm and 786 nm. A constant filling factor of 80% was used for both devices so as to create a fair comparison, with both device tested with ultra-pure laboratory water. Hence, we observe a reduction of 33% in the modulation depth at the lower wavelength range. This result corroborates the theoretical underpinning of the device mechanism discussed in the main text, whereby the core modulation mechanism stems from the enhanced ITO accumulation effects.

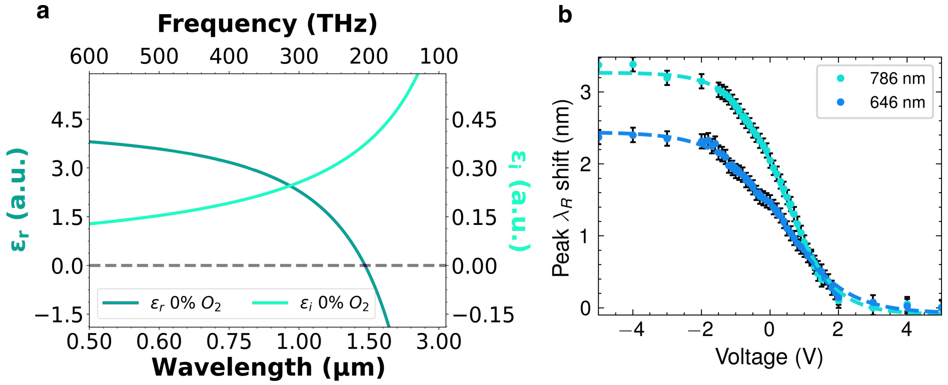


**Figure S4: ITO dispersive effects on modulation. a,** Experimentally determined dispersion curve for ITO[2] showing the real and imaginary trends across a large wavelength range. **b,** Modulation data for devices operating at 646 nm and 786 nm using a laboratory water fluid. The reduced modulation effects at visible wavelengths is clearly demonstrated.

# 5 Full phase data

Fig. S8 displays the full dataset for the results presented in Fig. 4. Here, region of interest (ROI) data can be seen for each voltage, with an example slice and moving average fit to clean the data. A sinusoidal fit for each moving average is shown in Fig. S8, along with the reference (0 V) data for comparison. Each phase value was extracted from the sinusoidal fit and calculated relative to the 0 V metasurface fringe. Four slices were taken for each inteferogram and averaged, and a total of four ROIs were used for each voltage to produce a statistically valid result and error (Figure S6).

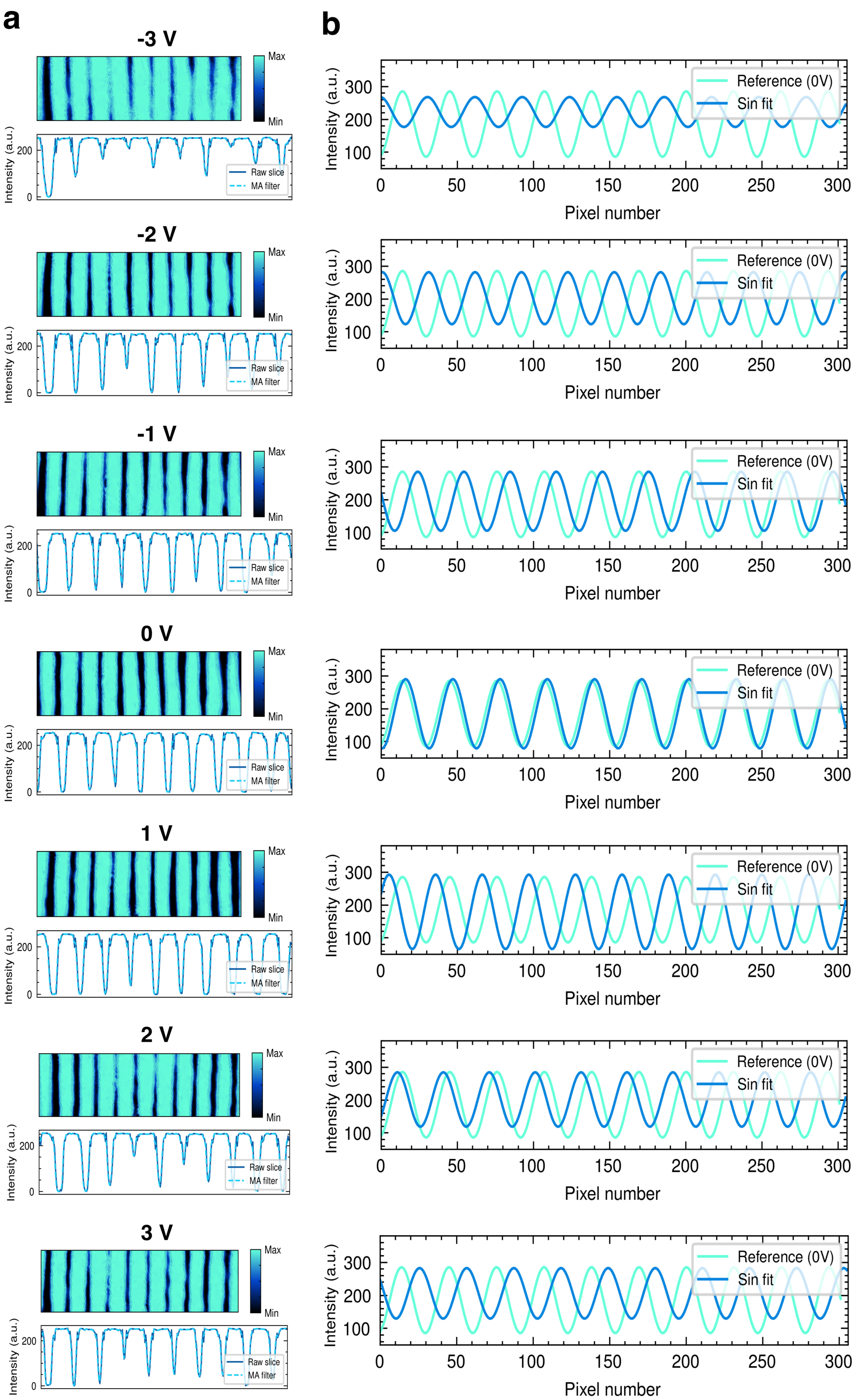


**Figure S5: Interference fringe data captured on a CMOS camera. a,** Shows the raw ROI data, with extracted slice data and a moving average fit shown underneath for data cleaning. **b,** Shows fitted sinusoidal waves for the reference and active signal from the tunable metasurface. The phase for each respective voltage was calculated from the fit with a corresponding error.

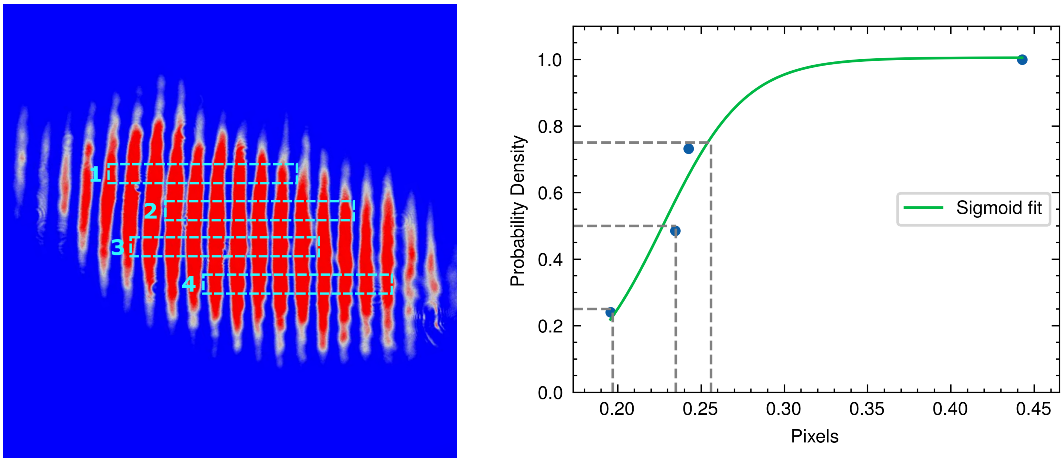


**a** **b**

**Figure S6: Phase data analysis. a,** Example interferogram data with ROIs highlighted across the image. **b,** Cumulative distribution function with a sigmoid fit for the statistical extraction of phase information from the ROIs. Lines are displayed at 25%, 50% and 75%, providing an average and the upper and lower bounds.

# 6 Broadband reflectance measurement setup

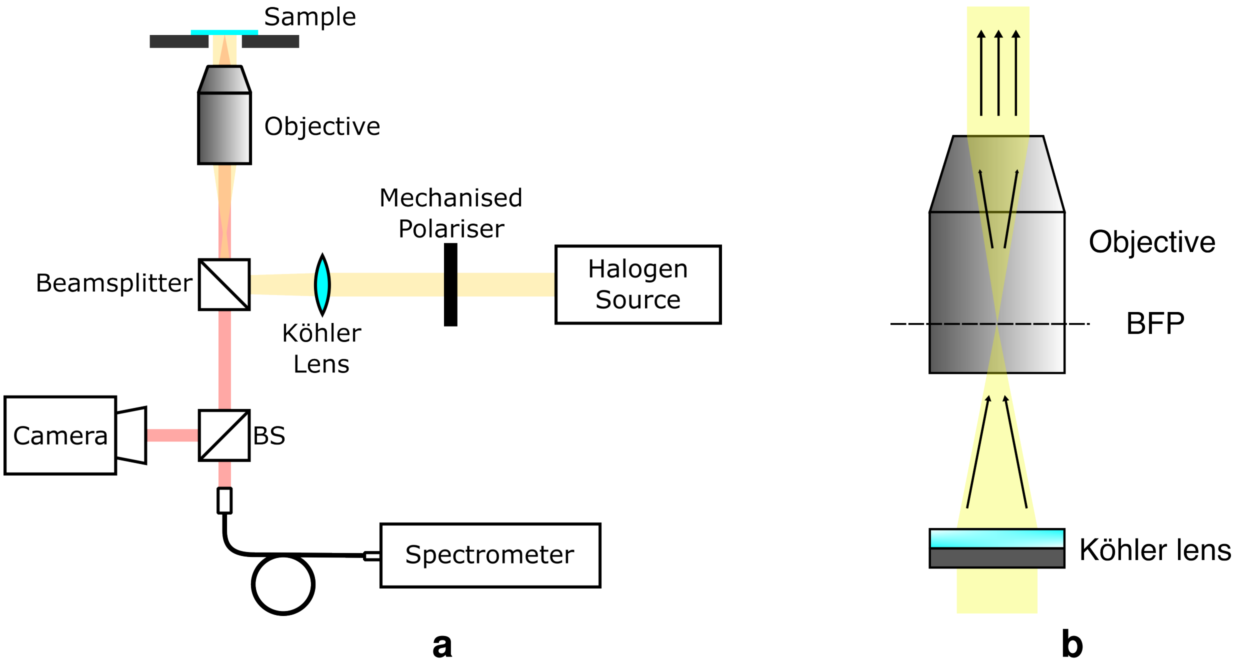


**Figure S7: Reflection optical setup a,** Diagram showing the full reflection setup used in the experiments. The setup consists of a broadband source, a polariser, beam splitter, objective, Köhler lens, camera and spectrometer. **b,** A schematic depicting Köhler illumination by using a lens to focus light into the back focal plane of the objective.

# 7 Phase measurement setup

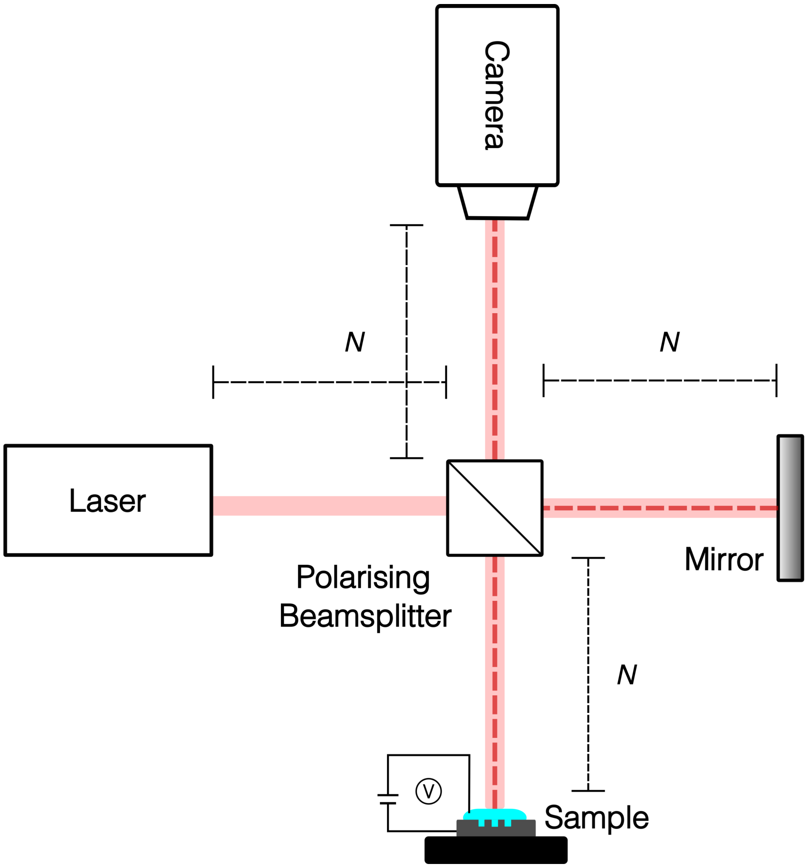


**Figure S8: Phase detection interferometer set up.** Here, the laser source is polarised and sent through an ND filter, before passing through a beam splitter where the signal is split between the mirror and device, before recombining on the camera sensor to form an interference pattern.

# 8 Switching speed measurements

To measure the modulation speed of the metasurface, we first illuminated the device with a polarised on-resonance laser. We next used a signal generator (GW INSTEK GFG-8219A) to apply an alternating square wave signal of ±1 V across the device, providing a cyclic variation of the phase and reflection as a function of time. The reflected signal was then fed into a photodetector (Thorlabs PDF10A/M, Si fW Sensitivity Fixed Gain Detector), to measure the response as a function of voltage on an oscilloscope. Fig. S9a depicts a schematic of the optical set up. Hence, by cycling through the frequency that was applied to the signal generator, we were able to accurately measure the reflection modulation as a function of frequency. The threshold was determined by the point at which the device modulation could no longer keep up with the applied signal. Modulation measurements for both laboratory water and an electrolyte solution (3 mol PBS solution) are shown in Fig. S9c,d, respectively, with no variation found between the two cases. Thus, our device comfortablely operates up to approximately 20 Hz, which is reasonable for an optofluidic device.

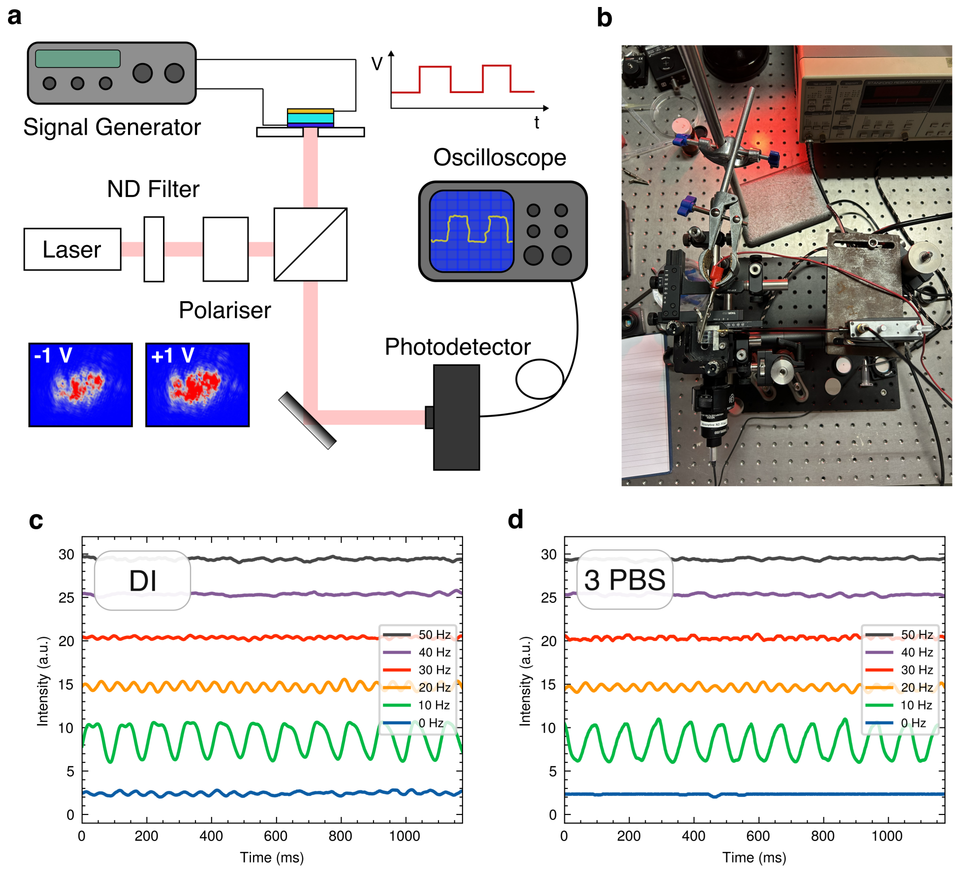


**Figure S9: Optofluidic metasurface switching speed. a,** Schematic depicting the optical set up used to measure the switching speed of the metasurface. The set up includes a signal generator, photodetector and oscilloscope for readout. **b,** Photograph of the optical set up. **c, d,** Switching speed measurements for laboratory water and an ionic solution. Both solutions sustain speeds up to 30 Hz (0.03 s).

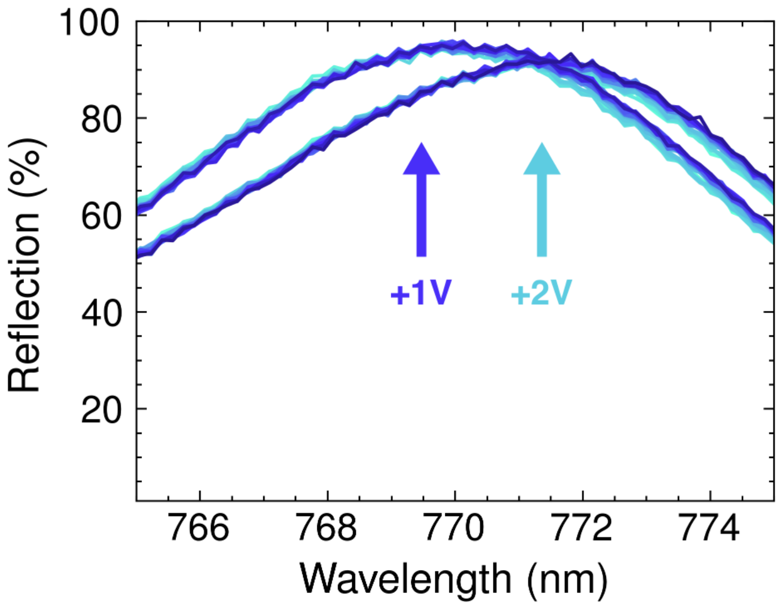


**a**

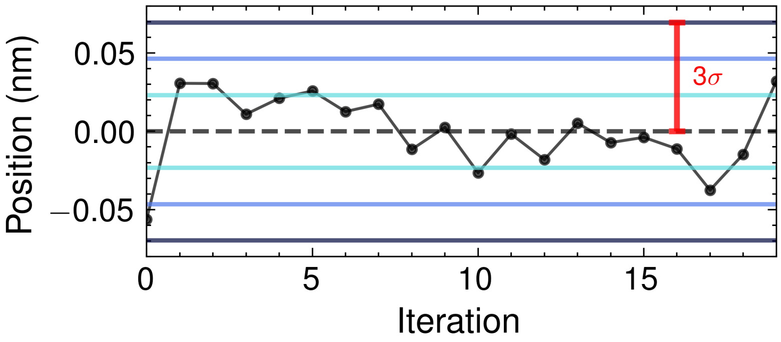


**b**

**Figure S10: Limit of detection measurements of the metasurface. a,** Repeated spectral measurements for 1 V and 2 V, **b**, Difference in peak positions for 20 iterations. The lines represented one, two and three standard deviations.

# 9 Limit of detection

A typical metric that is used to characterise a sensor is the limit of detection. The parameter essentially describes the smallest detectable shift that can be measured and is often given as three times the standard deviation ($\sigma$). Typically, one would take multiple measurements at a single resonant position and measure the fluctuation in peak position. However, for our device, we determined that a more suited approach was to activate

and deactivate the device (i.e., fluctuate between +1 V and +2 V) and record the relative difference in resonance position. The results are shown in Figure S10.

The colour gradient in Figure S10a displays different iterations (total of 20). We find the $3\sigma$ of the device to be 0.0696 nm. Dividing this value by the average sensitivity from the dilution series (882 nm/RIU) gives a limit of detection of $1.0 \times 10^{-4}$ RIU, in excellent agreement with other photonic crystal biosensors.[3] This value is already sufficient for detecting clinically relevant concentrations of biomolecules.

## 9.1 Achieving a $2\pi$ phase shift

The simulated results in Section 3 find a full $2\pi$ phase shift across a ±1 V range, whereas the experimental results achieve a phase shift of only $1.75\pi$ across ±3 V. A core contributing factor to this discrepancy is the reduced Q-factor of the experimental resonance, which decreases the gradient of the phase profile. The Q-factor was found to differ from simulation to experiment by a factor of 10. From SEM micrographs and optical measurements from plain SiN gratings, it is evident that the reduced Q is a result of the ITO. ITO exhibits a significant amount of surface roughness ($R_{RMS} \sim 4$ nm), which is the most likely culprit for the resonance broadening due to increased optical scattering in addition to the absorption loss from the high conductivity ITO.

Figure S11 shows a comparison of the simulated and experimental reflection and phase plots as a function of wavelength. The experimental phase data has been fitted with a sigmoid function. Clearly, the reduced experimental Q generates a less steep phase profile, meaning a larger degree of tuning is required to achieve the same phase shift. In theory, further increasing the applied bias would continue shifting the peak, meaning the full $2\pi$ phase profile would have been attainable. However, I found ±3 V to be a threshold voltage, after which the grating surface is fouled, rendering the device useless. Hence, the solution lies in either improving the ITO surface roughness to reduce scattering losses or increasing the filling factor (or more generally the effective index) to improve the Q-factor.

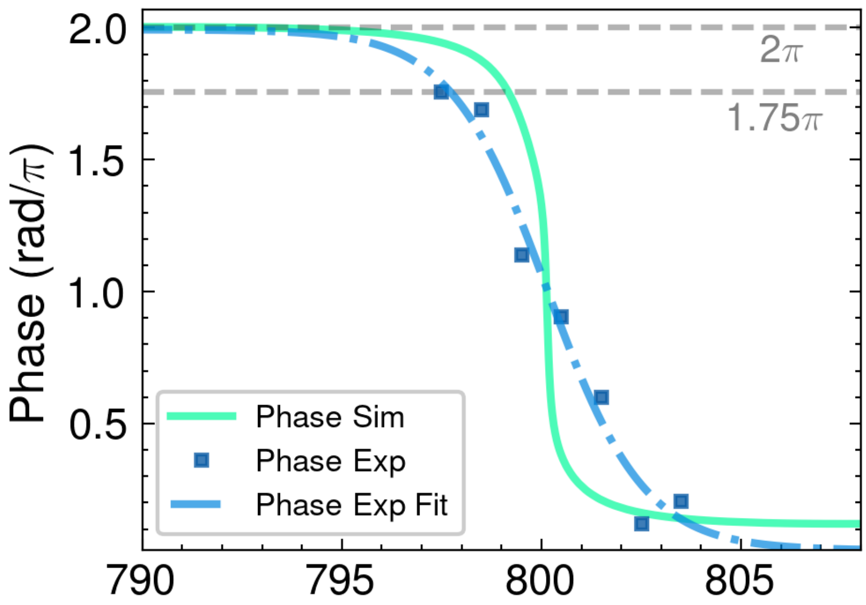


**Figure S11: Comparison of the simulated and experimental phase profiles of the device.** A difference of $0.25\pi$ is apparent due largely to the difference in the Q-factor of the resonances.

Firstly, increasing the filling factor is not a feasible approach. The fabrication would become too difficult as the grating grooves would be tens of nm. The sputtering of the ITO would also become too difficult, as the grooves would not fill with a conformal sidewall coating with a fill factor $> 80\%$, which is crucial for high-efficiency modulation. Moreover, the roughness could be readily improved by introducing a rapid thermal anneal (RTA) or using a quartz substrate to allow anneal temperatures greater than 500℃ to further improve the device imperfections and defects. The deposition conditions could also be altered to increase purity, such as reducing the deposition rate. Further, the simulated curve shifts approximately 5 nm, meanwhile, the experimental data was found to only shift approximately 3.5 nm across a larger voltage range. Hence, the amplification factor in the simulation likely overestimated the conductivity effects in the space charge region.

# References


(1) Feigenbaum, E.; Diest, K.; Atwater, H. A. Unity-Order Index Change in Transparent Conducting Oxides at Visible Frequencies. *Nano Letters* **2010**, *10*, 2111–2116.

(2) Blair, S. F. J.; Male, J. S.; Cavill, S. A.; Reardon, C. P.; Krauss, T. F. Photonic Characterisation of Indium Tin Oxide as a Function of Deposition Conditions. *Nanomaterials* **2023**, *13*, 1990, Number: 13.

(3) Estevez, M.; Alvarez, M.; Lechuga, L. Integrated optical devices for lab-on-a-chip biosensing applications. *Laser & Photonics Reviews* **2012**, *6*, 463–487, _eprint: https://onlinelibrary.wiley.com/doi/pdf/10.1002/lpor.201100025.
